# Supplementary material for: Optimal control approaches for combining medicines and mosquito control in tackling dengue
Source: R Soc Open Sci. 2020 Apr 22;7(4):181843. doi: 10.1098/rsos.181843 (PMC7211884; doi:10.1098/rsos.181843)
Supplement: S1 - Optimal control theory [file rsos181843supp1.pdf]

# S1 Appendix - Optimal Control Theory

Optimal control is a powerful approach for investigating complex dynamics subject to constraints. We provide a very brief overview of the key ideas of optimal control relevant to dengue disease management. In mathematical biology it is very common to consider a system whose behaviour can be explained by a series of ordinary differential equations (ODEs). In many of these cases, some of the variables may be externally controlled, for example in drug-interaction simulations the amount of drug administered is directly controlled. We call these **control variables**, and those that cannot be influenced are referred to as **state variables**. The goal of optimal control is then to determine, mathematically, the best choice of values for these control variables over time to achieve a certain aim. In the pharmacological example, one may wish to estimate the optimal amount of a drug administered to minimise tumour size while also minimising adverse side-effects [1].

Unsurprisingly, disease dynamics are readily amenable to optimal control problems; often the key epidemiological question is - what is the optimal choice of disease intervention measures (vaccines, vector-control methods) to minimise the number of infected individuals while also trying to minimise the economic cost of such measures. Traditional SIR networks are well formulated for optimal control approaches, and are commonly used in identifying optimal vaccination programmes [2], often in parallel with other methods and/or treatments [3]. One simple example is presented by Yusuf & Benyah (2012) [4].

To begin we define a system of ODEs to describe the behaviour of these state variables $x(t)$ that depends on the control variables $u(t)$:

$$\frac{\mathrm{d}x(t)}{\mathrm{d}t} = g(t, x(t), u(t)),$$

for some function $g$. We can also express, mathematically, what is wished to be minimised or maximised. This will be some expression of our state variables and control variables, as we will want to minimise a state (for example, the number of infected hosts), while also not letting the amount of control applied balloon beyond what is feasibly manageable. We express this desire as wishing to minimise (or perhaps maximise),

$$J(u) = \int_{t_0}^{t_1} f(t, x(t), u(t))\mathrm{d}t,$$

for some function $f$. Here $t_0$ and $t_1$ represent the start and finish of the time period we consider. Again we seek a particular optimal control variable, $u^*(t)$, and its corresponding optimal state, $x^*(t)$, that will minimise or maximise $J$.

The key result on which all of optimal control theory is built on is known as **Pontryagin's Maximum Principle** [5] [6].

We consider the Hamiltonian ($H$) as,

$$H(t, x(t), u(t), \lambda(t)) = f(t, x(t), u(t)) + \lambda(t) g(t, x(t), u(t)),$$

where $f$ and $g$ are as defined above and $\lambda(t)$ is a piecewise differentiable function known as the **adjoint variable**. Pontryagin's Maximum Principle then states that, if $u^*(t)$ and $x^*(t)$ are optimal such that they maximise (or minimise) $J(u)$, then there exists a particular adjoint variable $\lambda(t)$ such that,

$$H(t, x^*(t), u(t), \lambda(t)) \leq H(t, x^*(t), u^*(t), \lambda(t)),$$

for all control variables $u$ for all times $t$, if $\lambda(t)$ is such that,

$$\begin{aligned} \frac{\mathrm{d}\lambda}{\mathrm{d}t} &= -\frac{\partial H(t, x^*(t), u^*(t), \lambda(t))}{\partial x}, \\ \lambda(t_1) &= 0. \end{aligned}$$

Unsurprisingly this critical point is the optimal maximum. Note that the mathematical explanation follows the exact same procedure for minimisation problems, resulting only in an opposite inequality. As it is an optimum point, it is also the case that $\frac{\partial H}{\partial u} = 0$ at $u^*$. We formally present the three conditions that an optimal problem satisfies,

$$\begin{aligned} &\frac{\partial H}{\partial u} = 0 \text{ at } u^* && \text{(Optimality Condition)}, \\ &\frac{\mathrm{d}\lambda}{\mathrm{d}t} = -\frac{\partial H(t, x^*(t), u^*(t), \lambda(t))}{\partial x} && \text{(Adjoint Equations)}, \\ &\lambda(t_1) = 0 && \text{(Transversality Condition)}. \end{aligned}$$

For very simple problems this can be solved analytically, however for more realistic, and complex, problem formulations, the solution must be found numerically, via an iterative method. In general we consider some initial states, $u_0$, $x_0$ and $\lambda_0$. Usually we pick the zero function for all three as a starting point, but enforce the initial condition of the system $x(0)$ on $x_0$. Then take the following steps.

- Solve the system of equations $\dfrac{\mathrm{d}x_1(t)}{\mathrm{d}t} = g(t, x_0(t), u_0(t))$ using the initial condition of the system to obtain a new $x_1$.
- Solve the system of equations $\frac{\mathrm{d}\lambda_1}{\mathrm{d}t} = -\frac{\partial H(t, x_1(t), u_0(t), \lambda_0(t))}{\partial x}$ using the transversality condition $\lambda(t_1) = 0$ to produce a new $\lambda_1$.
- Using the optimality condition $\frac{\partial H}{\partial u} = 0$, $u$ can be re-arranged as an expression of $x$ and $\lambda$. Use this expression to build a new $u_1$ from $x_1$ and $\lambda_1$.
- Repeat these steps until a desired point of convergence.

# References


[1] R. Martin, Optimal control drug scheduling of cancer chemotherapy, Automatica 28 (6) (1992) 1113–1123.

[2] T. Kar, A. Batabyal, Stability analysis and optimal control of an sir epidemic model with vaccination, Biosystems 104 (2-3) (2011) 127–135.

[3] K. O. Okosun, R. Ouifki, N. Marcus, Optimal control analysis of a malaria disease transmission model that includes treatment and vaccination with waning immunity, Biosystems 106 (2-3) (2011) 136–145.

[4] T. T. Yusuf, F. Benyah, Optimal control of vaccination and treatment for an sir epidemiological model, World Journal of Modelling and Simulation 8 (3) (2012) 194–204.

[5] L. Pontryagin, V. Boltyanskii, R. Gamkrelidze, E. Mishchenko, The mathematical theory of optimal processes, CRC Press, 1962.

[6] S. Lenhart, J. T. Workman, Optimal control applied to biological models, CRC Press, 2007.
