# Supplementary material for: Optimal control approaches for combining medicines and mosquito control in tackling dengue
Source: R Soc Open Sci. 2020 Apr 22;7(4):181843. doi: 10.1098/rsos.181843 (PMC7211884; doi:10.1098/rsos.181843)
Supplement: S2 - The Hamiltonian [file rsos181843supp2.pdf]

S2 Appendix - The Hamiltonian

We present the Hamiltonian H used for our optimal control problem, for a general n settlements. Again, for notational ease, our adjoint functions will be written as λ_j^i, where i refers to the settlement of the associated state variable, and j to which of the 14 state variables it is associated with. All constants and variables are defined in Table 1 and Table 2, save for C_1, C_2 and C_3 which represent the cost values of treating dengue patients, providing vaccines and releasing sterile mosquitoes, respectively.

$$
\begin{aligned}
H =& C_1\left(\sum_{i=1}^{n} {I_i^A}^2 + {I_i^B}^2 + {I_i^{A_2}}^2 + {I_i^{B_2}}^2\right) + C_2\left(\sum_{i=1}^{n} {u_1^i}^2\right) + C_3\left(\sum_{i=1}^{n} {u_2^i}^2\right) \\
&+ \lambda_1^1\left(-\frac{S_1 a b_1 (X_1+Y_1)}{N_1+\Omega_1} - u_1^1 S_1\right) + \lambda_2^1\left(\frac{S_1 a b_1 X_1}{N_1+\Omega_1} - I_1^A(\alpha_1+\rho_1)\right) + \lambda_3^1\left(\rho_1 I_1^A - \eta R_1^A\right) \\
&+ \lambda_4^1\left(\eta R_1^A - \frac{S_1^{B_2} a b_2 Y_1}{N_1+\Omega_1}\right) + \lambda_5^1\left(\frac{S_1^{B_2} a b_2 Y_1}{N_1+\Omega_1} - I_1^{B_2}(\alpha_2+\rho_2)\right) + \lambda_6^1\left(\frac{S_1 a b_1 Y_1}{N_1+\Omega_1} - I_1^B(\alpha_1+\rho_1)\right) \\
&+ \lambda_7^1\left(\rho_1 I_1^B - \eta R_1^B + (1-\xi)u_1^1 S_1\right) + \lambda_8^1\left(\eta R_1^B - \frac{S_1^{A_2} a b_2 X_1}{N_1+\Omega_1}\right) + \lambda_9^1\left(\frac{S_1^{A_2} a b_2 X_1}{N_1+\Omega_1} - I_1^{A_2}(\alpha_2+\rho_2)\right) \\
&+ \lambda_{10}^1\left(\rho_2(I_1^{B_2} + I_1^{A_2}) + \xi u_1^1 S_1\right) + \lambda_{11}^1\left(g Z_1\left(\frac{Z_1 + (1-\epsilon)\psi_1 u_2^1}{Z_1 + \psi_1 u_2^1}\right) - J_1 \ln(1+\delta J_1) - \phi J_1\right) \\
&+ \lambda_{12}^1\left(\phi J_1 - \mu M_1 - \frac{a c M_1}{N_1+\Omega_1}\left(I_1^A + I_1^B + I_1^{A_2} + I_1^{B_2} + \sum_{i=2}^{n}\left(m_I(I_i^A + I_i^B) + m_{I_2}(I_i^{A_2} + I_i^{B_2})\right)\right)\right) \\
&+ \lambda_{13}^1\left(\frac{a c M_1}{N_1+\Omega_1}\left(I_1^A + I_1^{A_2} + \sum_{i=2}^{n}\left(m_I I_i^A + m_{I_2} I_i^{A_2}\right)\right) - \mu X_1\right) \\
&+ \lambda_{14}^1\left(\frac{a c M_1}{N_1+\Omega_1}\left(I_1^B + I_1^{B_2} + \sum_{i=2}^{n}\left(m_I I_i^B + m_{I_2} I_i^{B_2}\right)\right) - \mu Y_1\right) \\
&+ \sum_{i=2}^{n}\Bigg[\lambda_1^i\left(-(1-m_S)\frac{S_i a b_1(X_i+Y_i)}{N_i+\Omega_i} - m_S\frac{S_i a b_1 (X_1+Y_1)}{N_1+\Omega_1} - u_1^i S_i\right) \\
&+ \lambda_2^i\left((1-m_S)\frac{S_i a b_1 X_i}{N_i+\Omega_i} + m_S\frac{S_i a b_1 X_1}{N_1+\Omega_1} - I_i^A(\alpha_1+\rho_1)\right) + \lambda_3^i\left(\rho_1 I_i^A - \eta R_i^A\right) \\
&+ \lambda_4^i\left(\eta R_i^A - (1-m_R)\frac{S_i^{B_2} a b_2 Y_i}{N_i+\Omega_i} - m_R\frac{S_i^{B_2} a b_2 Y_1}{N_1+\Omega_1}\right) \\
&+ \lambda_5^i\left((1-m_R)\frac{S_i^{B_2} a b_2 Y_i}{N_i+\Omega_i} + m_R\frac{S_i^{B_2} a b_2 Y_1}{N_1+\Omega_1} - I_i^{B_2}(\alpha_2+\rho_2)\right) \\
&+ \lambda_6^i\left((1-m_S)\frac{S_i a b_1 Y_i}{N_i+\Omega_i} + m_S\frac{S_i a b_1 Y_1}{N_1+\Omega_1} - I_2^B(\alpha_1+\rho_1)\right) + \lambda_7^i\left(\rho_1 I_i^B - \eta R_i^B + (1-\xi)u_1^i S_i\right) \\
&+ \lambda_8^i\left(\eta R_i^B - (1-m_R)\frac{S_i^{A_2} a b_2 X_i}{N_i+\Omega_i} - m_R\frac{S_i^{A_2} a b_2 X_i}{N_1+\Omega_1}\right) \\
&+ \lambda_9^i\left((1-m_R)\frac{S_i^{A_2} a b_2 X_i}{N_i+\Omega_i} + m_R\frac{S_i^{A_2} a b_2 X_1}{N_1+\Omega_1} - I_i^{A_2}(\alpha_2+\rho_2)\right) + \lambda_{10}^i\left(\rho_2(I_i^{A_2} + I_i^{B_2}) + \xi u_1^i S_i\right) \\
&+ \lambda_{11}^i\left(g Z_i\left(\frac{Z_i}{Z_i+\psi_i u_2^i} + (1-\epsilon)\frac{\psi_i u_2^i}{Z_i+\psi_i u_2^i}\right) - J_i \ln(1+\delta J_i) - \phi J_i\right) \\
&+ \lambda_{12}^i\left(\phi J_i - \mu M_i - \frac{a c M_i}{N_i+\Omega_i}\left((I_i^A + I_i^B)(1-m_I) + (I_i^{A_2} + I_i^{B_2})(1-m_{I_2})\right)\right) \\
&+ \lambda_{13}^i\left(\frac{a c M_i}{N_i+\Omega_i}\left(I_i^A(1-m_I) + I_i^{A_2}(1-m_{I_2})\right) - \mu X_i\right) \\
&+ \lambda_{14}^i\left(\frac{a c M_i}{N_i+\Omega_i}\left(I_i^B(1-m_I) + I_i^{B_2}(1-m_{I_2})\right) - \mu Y_i\right)\Bigg]
\end{aligned}
$$
