# Supplementary material for: Optimal control approaches for combining medicines and mosquito control in tackling dengue
Source: R Soc Open Sci. 2020 Apr 22;7(4):181843. doi: 10.1098/rsos.181843 (PMC7211884; doi:10.1098/rsos.181843)
Supplement: S3 - Adjoint equation [file rsos181843supp3.pdf]

S3 Appendix - Adjoint Equations

We present two sets of adjoint equations. One for the central city settlement, and one for the outer town settlements.

Central City Settlement Adjoint Equations

We begin by initially defining a function, F, of state and adjoint variables that appears throughout the adjoint equations for further notational ease.

$$
\begin{aligned}
F := &-\lambda_1^1\left(\frac{S_1ab_1(X_1+Y_1)}{(N_1+\Omega_1)^2}\right)+\lambda_2^1\left(\frac{S_1ab_1X_1}{(N_1+\Omega_1)^2}\right)-\lambda_4^1\left(\frac{S_1^{B_2}ab_2Y_1}{(N_1+\Omega_1)^2}\right)\\
&+\lambda_5^1\left(\frac{S_1^{B_2}ab_2Y_1}{(N_1+\Omega_1)^2}\right)+\lambda_6^1\left(\frac{S_1ab_1Y_1}{(N_1+\Omega_1)^2}\right)-\lambda_8^1\left(\frac{S_1^{A_2}ab_2X_1}{(N_1+\Omega_1)^2}\right)\\
&+\lambda_9^1\left(\frac{S_1^{A_2}ab_2X_1}{(N_1+\Omega_1)^2}\right)-\lambda_{12}^1\left(\frac{acM_1}{(N_1+\Omega_1)^2}(I_1^A+I_1^B+I_1^{A_2}+I_1^{B_2})\right)\\
&+\lambda_{13}^1\left(\frac{acM_1}{(N_1+\Omega_1)^2}(I_1^A+I_1^{A_2})\right)+\lambda_{14}^1\left(\frac{acM_1}{(N_1+\Omega_1)^2}(I_1^B+I_1^{B_2})\right)\\
&+\sum_{i=2}^{n}\Bigg(-\lambda_{12}^1\left(\frac{acM_1}{(N_1+\Omega_1)^2}\left(m_I(I_i^A+I_i^B)+m_{I_2}(I_i^{A_2}+I_i^{B_2})\right)\right)\\
&+\lambda_{13}^1\left(\frac{acM_1}{(N_1+\Omega_1)^2}(m_II_i^A+m_{I_2}I_i^{A_2})\right)+\lambda_{14}^1\left(\frac{acM_1}{(N_1+\Omega_1)^2}(m_II_i^B+m_{I_2}I_i^{B_2})\right)\\
&-\lambda_1^i\left(\frac{m_SS_iab_1(X_1+Y_1)}{(N_1+\Omega_1)^2}\right)+\lambda_2^i\left(\frac{m_SS_iab_1X_1}{(N_1+\Omega_1)^2}\right)-\lambda_4^i\left(\frac{m_RS_i^{B_2}ab_2Y_1}{(N_1+\Omega_1)^2}\right)\\
&+\lambda_5^i\left(\frac{m_RS_i^{B_2}ab_2Y_1}{(N_1+\Omega_1)^2}\right)+\lambda_6^i\left(\frac{m_SS_iab_1Y_1}{(N_1+\Omega_1)^2}\right)-\lambda_8^i\left(\frac{m_RS_i^{A_2}ab_2X_1}{(N_1+\Omega_1)^2}\right)\\
&+\lambda_9^i\left(\frac{m_RS_i^{A_2}ab_2X_1}{(N_1+\Omega_1)^2}\right)\Bigg).
\end{aligned}
$$

We now state the actual adjoint equations for the city settlement.

$$
\begin{aligned}
\frac{\mathrm{d}\lambda_1^1}{\mathrm{d}t} =&F + \lambda_1^1\left(\frac{ab_1(X_1+Y_1)}{N_1+\Omega_1}+u_1^1\right) - \lambda_2^1\left(\frac{ab_1X_1}{N_1+\Omega_1}\right) - \lambda_6^1\left(\frac{ab_1Y_1}{N_1+\Omega_1}\right)\\
&-\lambda_7^1(1-\xi)u_1^1 - \lambda_{10}^1\xi u_1^1\\
\frac{\mathrm{d}\lambda_2^1}{\mathrm{d}t} =&F - 2C_1I_1^A + \lambda_2^1(\alpha_1+\rho_1) - \lambda_3^1\rho_1 + \lambda_{12}^1\left(\frac{acM_1}{N_1+\Omega_1}\right) - \lambda_{13}^1\left(\frac{acM_1}{N_1+\Omega_1}\right)\\
\frac{\mathrm{d}\lambda_3^1}{\mathrm{d}t} =&F + \lambda_3^1\eta - \lambda_4^1\eta\\
\frac{\mathrm{d}\lambda_4^1}{\mathrm{d}t} =&F + \lambda_4^1\left(\frac{ab_2Y_1}{N_1+\Omega_1}\right) - \lambda_5^1\left(\frac{ab_2Y_1}{N_1+\Omega_1}\right)\\
\frac{\mathrm{d}\lambda_5^1}{\mathrm{d}t} =&F - 2C_1I_1^{B_2} + \lambda_5^1(\alpha_2+\rho_2) - \lambda_{10}^1\rho_2 + \lambda_{12}^1\left(\frac{acM_1}{N_1+\Omega_1}\right) - \lambda_{14}^1\left(\frac{acM_1}{N_1+\Omega_1}\right)\\
\frac{\mathrm{d}\lambda_6^1}{\mathrm{d}t} =&F - 2C_1I_1^B + \lambda_6^1(\alpha_1+\rho_1) - \lambda_7^1\rho_1 + \lambda_{12}^1\left(\frac{acM_1}{N_1+\Omega_1}\right) - \lambda_{14}^1\left(\frac{acM_1}{N_1+\Omega_1}\right)\\
\frac{\mathrm{d}\lambda_7^1}{\mathrm{d}t} =&F + \lambda_7^1\eta - \lambda_8^1\eta\\
\frac{\mathrm{d}\lambda_8^1}{\mathrm{d}t} =&F + \lambda_8^1\left(\frac{ab_2X_1}{N_1+\Omega_1}\right) - \lambda_9^1\left(\frac{ab_2X_1}{N_1+\Omega_1}\right)\\
\frac{\mathrm{d}\lambda_9^1}{\mathrm{d}t} =&F - 2C_1I_1^{A_2} + \lambda_9^1(\alpha_2+\rho_2) - \lambda_{10}^1\rho_2 + \lambda_{12}^1\left(\frac{acM_1}{N_1+\Omega_1}\right) - \lambda_{13}^1\left(\frac{acM_1}{N_1+\Omega_1}\right)\\
\frac{\mathrm{d}\lambda_{10}^1}{\mathrm{d}t} =&F\\
\frac{\mathrm{d}\lambda_{11}^1}{\mathrm{d}t} =&\lambda_{11}^1\left(\phi + \frac{\delta J_1}{1+\delta J_1} + \ln(1+\delta J_1)\right) - \lambda_{12}^1\phi\\
\frac{\mathrm{d}\lambda_{12}^1}{\mathrm{d}t} =&-\lambda_{11}^1 g\left(\frac{Z_1+\psi_1u_2^1(1-\epsilon)}{Z_1+\psi_1u_2^1} + \frac{Z_1\epsilon\psi_1u_2^1}{(Z_1+\psi_1u_2^1)^2}\right)\\
&+\lambda_{12}^1\left(\mu + \frac{ac}{N_1+\Omega_1}\left(I_1^A + I_1^B + I_1^{A_2} + I_1^{B_2} + \sum_{i=2}^{n}\left(m_I(I_i^A+I_i^B) + m_{I_2}(I_i^{A_2}+I_i^{B_2})\right)\right)\right)\\
&-\lambda_{13}^1\left(\frac{ac}{N_1+\Omega_1}\left(I_1^A + I_1^{A_2} + \sum_{i=2}^{n}\left(m_I I_i^A + m_{I_2}I_i^{A_2}\right)\right)\right)\\
&-\lambda_{14}^1\left(\frac{ac}{N_1+\Omega_1}\left(I_1^B + I_1^{B_2} + \sum_{i=2}^{n}\left(m_I I_i^B + m_{I_2}I_i^{B_2}\right)\right)\right)\\
\frac{\mathrm{d}\lambda_{13}^1}{\mathrm{d}t} =&\lambda_1^1\left(\frac{S_1ab_1}{N_1+\Omega_1}\right) - \lambda_2^1\left(\frac{S_1ab_1}{N_1+\Omega_1}\right) + \lambda_8^1\left(\frac{S_1^{A_2}ab_2}{N_1+\Omega_1}\right) - \lambda_9^1\left(\frac{S_1^{A_2}ab_2}{N_1+\Omega_1}\right)\\
&-\lambda_{11}^1 g\left(\frac{Z_1+\psi_1u_2^1(1-\epsilon)}{Z_1+\psi_1u_2^1} + \frac{Z_1\epsilon\psi_1u_2^1}{(Z_1+\psi_1u_2^1)^2}\right) + \lambda_{13}^1\mu\\
&+\sum_{i=2}^{n}\left(\lambda_1^i\left(\frac{m_SS_iab_1}{N_1+\Omega_1}\right) - \lambda_2^i\left(\frac{m_SS_iab_1}{N_1+\Omega_1}\right) + \lambda_8^i\left(\frac{m_RS_i^{A_2}ab_2}{N_1+\Omega_1}\right) - \lambda_9^i\left(\frac{m_RS_i^{A_2}ab_2}{N_1+\Omega_1}\right)\right)
\end{aligned}
$$

$$
\begin{aligned}
\frac{\mathrm{d}\lambda_{14}^1}{\mathrm{d}t} =&\lambda_1^1\left(\frac{S_1ab_1}{N_1+\Omega_1}\right) - \lambda_6^1\left(\frac{S_1ab_1}{N_1+\Omega_1}\right) + \lambda_4^1\left(\frac{S_1^{B_2}ab_2}{N_1+\Omega_1}\right) - \lambda_5^1\left(\frac{S_1^{B_2}ab_2}{N_1+\Omega_1}\right)\\
&-\lambda_{11}^1 g\left(\frac{Z_1+\psi_1u_2^1(1-\epsilon)}{Z_1+\psi_1u_2^1} + \frac{Z_1\epsilon\psi_1u_2^1}{(Z_1+\psi_1u_2^1)^2}\right) + \lambda_{14}^1\mu\\
&+\sum_{i=2}^{n}\left(\lambda_1^i\left(\frac{m_SS_iab_1}{N_1+\Omega_1}\right) - \lambda_6^i\left(\frac{m_SS_iab_1}{N_1+\Omega_1}\right) + \lambda_4^i\left(\frac{m_RS_i^{B_2}ab_2}{N_1+\Omega_1}\right) - \lambda_5^i\left(\frac{m_RS_i^{B_2}ab_2}{N_1+\Omega_1}\right)\right)
\end{aligned}
$$

Outer Town Settlement Adjoint Equations

We now present the adjoint equations for the town settlements, $\frac{\mathrm{d}\lambda_j^i}{\mathrm{d}t}$ for all j and for all $i \geq 2$. We start like before by initially defining a function, G_j^i, of state and adjoint variables that appear throughout the adjoint equations for further notational ease. This function varies in one small way throughout the equations. We define a new constant, $\hat{m}_j$. $\hat{m}_j = m_S$ for $j = 1$. $\hat{m}_j = m_I$ for $j = 2$. $\hat{m}_j = m_R$ for $j = 3$. $\hat{m}_j = m_{I_2}$ for $j = 4$. j will be specified by a subscript on the function itself, G_j^i.

$$
\begin{aligned}
G_j^i = &- \lambda_1^1 \left(\frac{\hat{m}_j S_1 ab_1(X_1 + Y_1)}{(N_1 + \Omega_1)^2} \right) + \lambda_2^1 \left(\frac{\hat{m}_j S_1 ab_1 X_1}{(N_1 + \Omega_1)^2} \right) + \lambda_6^1 \left(\frac{\hat{m}_j S_1 ab_1 Y_1}{(N_1 + \Omega_1)^2} \right) \\
&- \lambda_4^1 \left(\frac{\hat{m}_j S_1^{B_2} ab_2 Y_1}{(N_1 + \Omega_1)^2} \right) + \lambda_5^1 \left(\frac{\hat{m}_j S_1^{B_2} ab_2 Y_1}{(N_1 + \Omega_1)^2} \right) - \lambda_8^1 \left(\frac{\hat{m}_j S_1^{A_2} ab_2 X_1}{(N_1 + \Omega_1)^2} \right) \\
&+ \lambda_9^1 \left(\frac{\hat{m}_j S_1^{A_2} ab_2 X_1}{(N_1 + \Omega_1)^2} \right) - \lambda_{12}^1 \left(\frac{\hat{m}_j acM_1}{(N_1 + \Omega_1)^2} (I_1^A + I_1^B + I_1^{A_2} + I_1^{B_2}) \right) \\
&+ \lambda_{13}^1 \left(\frac{\hat{m}_j acM_1}{(N_1 + \Omega_1)^2} (I_1^A + I_1^{A_2}) \right) + \lambda_{14}^1 \left(\frac{\hat{m}_j acM_1}{(N_1 + \Omega_1)^2} (I_1^B + I_1^{B_2}) \right) \\
&- \lambda_1^i \left((1 - m_S)(1 - \hat{m}_j) \frac{S_i ab_1(X_i + Y_i)}{(N_i + \Omega_i)^2} \right) + \lambda_2^i \left((1 - m_S)(1 - \hat{m}_j) \frac{S_i ab_1 X_i}{(N_i + \Omega_i)^2} \right) \\
&- \lambda_4^i \left((1 - m_R)(1 - \hat{m}_j) \frac{S_i^{B_2} ab_2 Y_i}{(N_i + \Omega_i)^2} \right) + \lambda_5^i \left((1 - m_R)(1 - \hat{m}_j) \frac{S_i^{B_2} ab_2 Y_i}{(N_i + \Omega_i)^2} \right) \\
&+ \lambda_6^i \left((1 - m_S)(1 - \hat{m}_j) \frac{S_i ab_1 Y_i}{(N_i + \Omega_i)^2} \right) - \lambda_8^i \left((1 - m_R)(1 - \hat{m}_j) \frac{S_i^{A_2} ab_2 X_i}{(N_i + \Omega_i)^2} \right) \\
&+ \lambda_9^i \left((1 - m_R)(1 - \hat{m}_j) \frac{S_i^{A_2} ab_2 X_i}{(N_i + \Omega_i)^2} \right) \\
&- \lambda_{12}^i \left((1 - \hat{m}_j) \frac{acM_i}{(N_i + \Omega_i)^2} \left((I_i^A + I_i^B)(1 - m_I) + (I_i^{A_2} + I_i^{B_2})(1 - m_{I_2}) \right) \right) \\
&+ \lambda_{13}^i \left((1 - \hat{m}_j) \frac{acM_i}{(N_i + \Omega_i)^2} \left(I_i^A (1 - m_I) + I_i^{A_2} (1 - m_{I_2}) \right) \right) \\
&+ \lambda_{14}^i \left((1 - \hat{m}_j) \frac{acM_i}{(N_i + \Omega_i)^2} \left(I_i^B (1 - m_I) + I_i^{B_2} (1 - m_{I_2}) \right) \right) \\
&+ \sum_{k=2}^{n} \left(-\lambda_{12}^1 \left(\frac{\hat{m}_j acM_1}{(N_1 + \Omega_1)^2} \left(m_I (I_k^A + I_k^B) + m_{I_2} (I_k^{A_2} + I_k^{B_2}) \right) \right) \right. \\
&+ \lambda_{13}^1 \left(\frac{\hat{m}_j acM_1}{(N_1 + \Omega_1)^2} \left(m_I I_k^A + m_{I_2} I_k^{A_2} \right) \right) + \lambda_{14}^1 \left(\frac{\hat{m}_j acM_1}{(N_1 + \Omega_1)^2} \left(m_I I_k^B + m_{I_2} I_k^{B_2} \right) \right) \\
&- \lambda_1^k \left(\frac{\hat{m}_j m_S S_k ab_1 (X_1 + Y_1)}{(N_1 + \Omega_1)^2} \right) + \lambda_2^k \left(\frac{\hat{m}_j m_S S_k ab_1 X_1}{(N_1 + \Omega_1)^2} \right) + \lambda_6^k \left(\frac{\hat{m}_j m_S S_k ab_1 Y_1}{(N_1 + \Omega_1)^2} \right) \\
&- \lambda_4^k \left(\frac{\hat{m}_j m_R S_k^{B_2} ab_2 Y_1}{(N_1 + \Omega_1)^2} \right) + \lambda_5^k \left(\frac{\hat{m}_j m_R S_k^{B_2} ab_2 Y_1}{(N_1 + \Omega_1)^2} \right) - \lambda_8^k \left(\frac{\hat{m}_j m_R S_k^{A_2} ab_2 X_1}{(N_1 + \Omega_1)^2} \right) \\
&\left. + \lambda_9^k \left(\frac{\hat{m}_j m_R S_k^{A_2} ab_2 X_1}{(N_1 + \Omega_1)^2} \right) \right).
\end{aligned}
$$

We now present the adjoint equations for the outer town settlements.

$$
\begin{aligned}
\frac{\mathrm{d}\lambda_1^i}{\mathrm{d}t} =& G_1^i + \lambda_1^i \left((1-m_S)\frac{ab_1(X_i+Y_i)}{N_i+\Omega_i} + \frac{m_S ab_1(X_1+Y_1)}{N_1+\Omega_1} + u_1^i \right) \\
&- \lambda_2^i \left((1-m_S)\frac{ab_1 X_i}{N_i+\Omega_i} + \frac{m_S ab_1 X_1}{N_1+\Omega_1} \right) - \lambda_6^i \left((1-m_S)\frac{ab_1 Y_i}{N_i+\Omega_i} + \frac{m_S ab_1 Y_1}{N_1+\Omega_1} \right) \\
&- \lambda_7^i (1-\xi) u_1^i - \lambda_{10}^i \xi u_1^i \\
\frac{\mathrm{d}\lambda_2^i}{\mathrm{d}t} =& G_2^i - 2C_1 I_i^A + \lambda_{12}^1 \left(m_I \frac{acM_1}{N_1+\Omega_1} \right) - \lambda_{13}^1 \left(m_I \frac{acM_1}{N_1+\Omega_1} \right) + \lambda_2^i(\alpha_1+\rho_1) \\
&- \lambda_3^i \rho_1 + \lambda_{12}^i \left((1-m_I)\frac{acM_i}{N_i+\Omega_i} \right) - \lambda_{13}^i \left((1-m_I)\frac{acM_i}{N_i+\Omega_i} \right) \\
\frac{\mathrm{d}\lambda_3^i}{\mathrm{d}t} =& G_3^i + \lambda_3^i \eta - \lambda_4^i \eta \\
\frac{\mathrm{d}\lambda_4^i}{\mathrm{d}t} =& G_3^i + \lambda_4^i \left((1-m_R)\frac{ab_2 Y_i}{N_i+\Omega_i} + \frac{m_R ab_2 Y_1}{N_1+\Omega_1} \right) - \lambda_5^i \left((1-m_R)\frac{ab_2 Y_i}{N_i+\Omega_i} + \frac{m_R ab_2 Y_1}{N_1+\Omega_1} \right) \\
\frac{\mathrm{d}\lambda_5^i}{\mathrm{d}t} =& G_4^i - 2C_1 I_i^{B_2} + \lambda_{12}^1 \left(m_{I_2} \frac{acM_1}{N_1+\Omega_1} \right) - \lambda_{14}^1 \left(m_{I_2} \frac{acM_1}{N_1+\Omega_1} \right) + \lambda_5^i(\alpha_2+\rho_2) \\
&- \lambda_{10}^i \rho_2 + \lambda_{12}^i \left((1-m_{I_2})\frac{acM_i}{N_i+\Omega_i} \right) - \lambda_{14}^i \left((1-m_{I_2})\frac{acM_i}{N_i+\Omega_i} \right) \\
\frac{\mathrm{d}\lambda_6^i}{\mathrm{d}t} =& G_2^i - 2C_1 I_i^B + \lambda_{12}^1 \left(m_I \frac{acM_1}{N_1+\Omega_1} \right) - \lambda_{14}^1 \left(m_I \frac{acM_1}{N_1+\Omega_1} \right) + \lambda_6^i(\alpha_1+\rho_1) \\
&- \lambda_7^i \rho_1 + \lambda_{12}^i \left((1-m_I)\frac{acM_i}{N_i+\Omega_i} \right) - \lambda_{14}^i \left((1-m_I)\frac{acM_i}{N_i+\Omega_i} \right) \\
\frac{\mathrm{d}\lambda_7^i}{\mathrm{d}t} =& G_3^i + \lambda_7^i \eta - \lambda_8^i \eta \\
\frac{\mathrm{d}\lambda_8^i}{\mathrm{d}t} =& G_3^i + \lambda_8^i \left((1-m_R)\frac{ab_2 X_i}{N_i+\Omega_i} + \frac{m_R ab_2 X_1}{N_1+\Omega_1} \right) - \lambda_9^i \left((1-m_R)\frac{ab_2 X_i}{N_i+\Omega_i} + \frac{m_R ab_2 X_1}{N_1+\Omega_1} \right) \\
\frac{\mathrm{d}\lambda_9^i}{\mathrm{d}t} =& G_4^i - 2C_1 I_i^{A_2} + \lambda_{12}^1 \left(m_{I_2} \frac{acM_1}{N_1+\Omega_1} \right) - \lambda_{13}^1 \left(m_{I_2} \frac{acM_1}{N_1+\Omega_1} \right) + \lambda_9^i(\alpha_2+\rho_2) \\
&- \lambda_{10}^i \rho_2 + \lambda_{12}^i \left((1-m_{I_2})\frac{acM_i}{N_i+\Omega_i} \right) - \lambda_{13}^i \left((1-m_{I_2})\frac{acM_i}{N_i+\Omega_i} \right) \\
\frac{\mathrm{d}\lambda_{10}^i}{\mathrm{d}t} =& G_3^i \\
\frac{\mathrm{d}\lambda_{11}^i}{\mathrm{d}t} =& \lambda_{11}^i \left(\phi + \frac{\delta J_i}{1+\delta J_i} + \ln(1+\delta J_i) \right) - \lambda_{12}^i \phi \\
\frac{\mathrm{d}\lambda_{12}^i}{\mathrm{d}t} =& - \lambda_{11}^i g \left(\frac{Z_i + \psi_i u_2^i (1-\epsilon)}{Z_i + \psi_i u_2^i} + \frac{Z_i \epsilon \psi_i u_2^i}{(Z_i+\psi_i u_2^i)^2} \right) \\
&+ \lambda_{12}^i \left(\mu + \frac{ac}{N_i+\Omega_i} \left((1-m_I)(I_i^A + I_i^B) + (1-m_{I_2})(I_i^{A_2} + I_i^{B_2}) \right) \right) \\
&- \lambda_{13}^i \left(\frac{ac}{N_i+\Omega_i} \left((1-m_I) I_i^A + (1-m_{I_2}) I_i^{A_2} \right) \right) \\
&- \lambda_{14}^i \left(\frac{ac}{N_i+\Omega_i} \left((1-m_I) I_i^B + (1-m_{I_2}) I_i^{B_2} \right) \right) \\
\frac{\mathrm{d}\lambda_{13}^i}{\mathrm{d}t} =& \lambda_1^i \left((1-m_S)\frac{S_i ab_1}{N_i+\Omega_i} \right) - \lambda_2^i \left((1-m_S)\frac{S_i ab_1}{N_i+\Omega_i} \right) + \lambda_8^i \left((1-m_R)\frac{S_i^{A_2} ab_2}{N_i+\Omega_i} \right) \\
&- \lambda_9^i \left((1-m_R)\frac{S_i^{A_2} ab_2}{N_i+\Omega_i} \right) - \lambda_{11}^i g \left(\frac{Z_i + \psi_i u_2^i (1-\epsilon)}{Z_i + \psi_i u_2^i} + \frac{Z_i \epsilon \psi_i u_2^i}{(Z_i+\psi_i u_2^i)^2} \right) + \lambda_{13}^i \mu \\
\frac{\mathrm{d}\lambda_{14}^i}{\mathrm{d}t} =& \lambda_1^i \left((1-m_S)\frac{S_i ab_1}{N_i+\Omega_i} \right) - \lambda_6^i \left((1-m_S)\frac{S_i ab_1}{N_i+\Omega_i} \right) + \lambda_4^i \left((1-m_R)\frac{S_i^{B_2} ab_2}{N_i+\Omega_i} \right) \\
&- \lambda_5^i \left((1-m_R)\frac{S_i^{B_2} ab_2}{N_i+\Omega_i} \right) - \lambda_{11}^i g \left(\frac{Z_i + \psi_i u_2^i (1-\epsilon)}{Z_i + \psi_i u_2^i} + \frac{Z_i \epsilon \psi_i u_2^i}{(Z_i+\psi_i u_2^i)^2} \right) + \lambda_{14}^i \mu
\end{aligned}
$$
