# Supplementary figures and images for: Optimal control approaches for combining medicines and mosquito control in tackling dengue
Source: R Soc Open Sci. 2020 Apr 22;7(4):181843. doi: 10.1098/rsos.181843 (PMC7211884; doi:10.1098/rsos.181843)

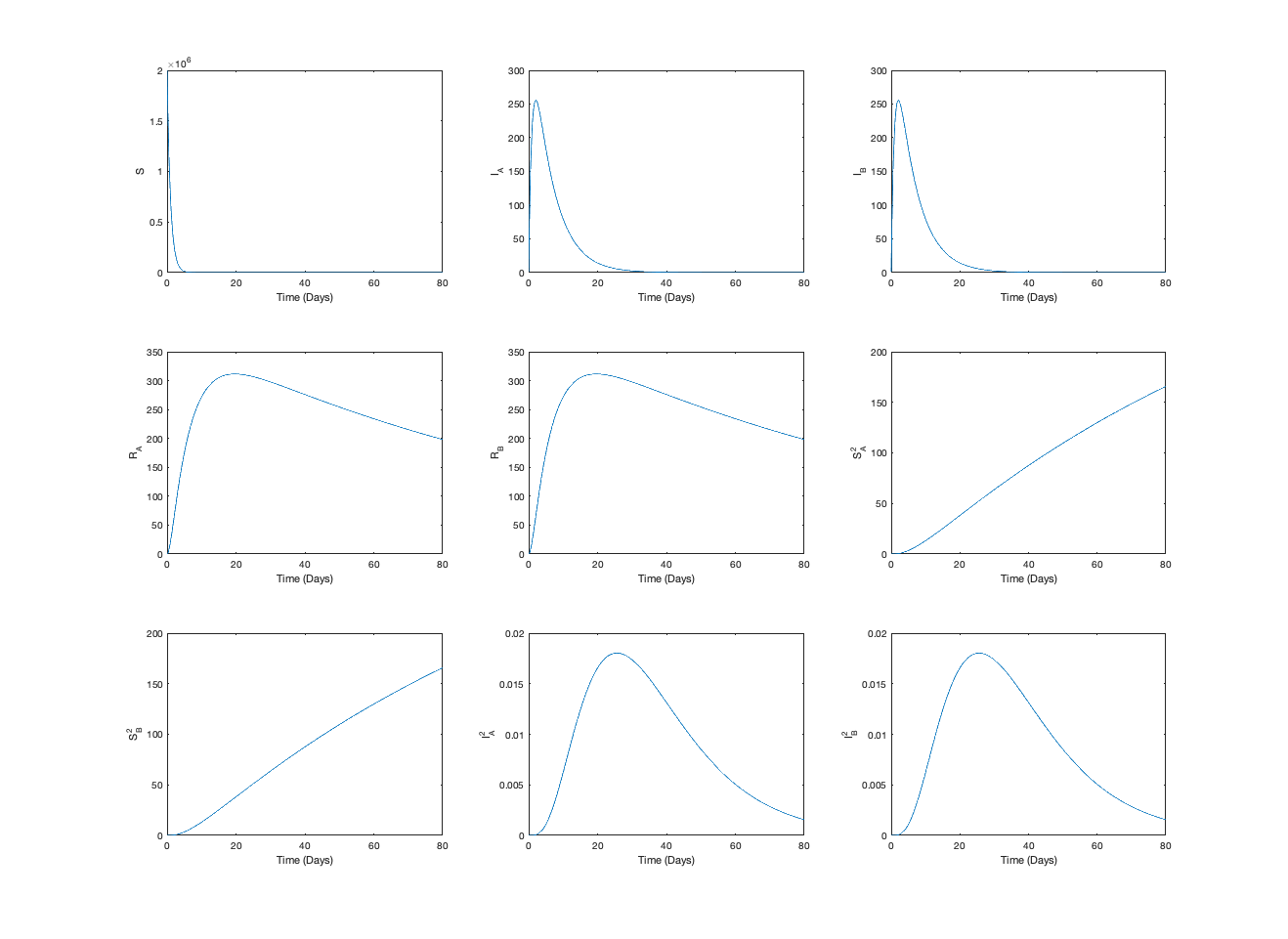

Supplement: S4 - Model Dynamics [file rsos181843supp4.tif]
